# Partition-based optimization model for generative anatomy modeling language (POM-GAML)

**DOI:** 10.1186/s12859-019-2626-7

**Published:** 2019-03-14

**Authors:** Doga Demirel, Berk Cetinsaya, Tansel Halic, Sinan Kockara, Shahryar Ahmadi

**Affiliations:** 1 Department of Computer Science, University of Arkansas at Little Rock, Little Rock, AR USA; 2 Department of Computer Science, University of Central Arkansas, Conway, AR USA; 3 Department of Orthopedic Surgery, University of Arkansas for Medical Sciences, Little Rock, AR USA

**Keywords:** Modeling language for human anatomy, Arthroscopic rotator cuff, Virtual human anatomy, Nonlinear programming, Partition-based optimization

## Abstract

**Background:**

This paper presents a novel approach for Generative Anatomy Modeling Language (GAML). This approach automatically detects the geometric partitions in 3D anatomy that in turn speeds up integrated non-linear optimization model in GAML for 3D anatomy modeling with constraints (e.g. joints). This integrated non-linear optimization model requires the exponential execution time. However, our approach effectively computes the solution for non-linear optimization model and reduces computation time from exponential to linear time. This is achieved by grouping the 3D geometric constraints into communities.

**Methods:**

Various community detection algorithms (k-means clustering, Clauset Newman Moore, and Density-Based Spatial Clustering of Applications with Noise) were used to find communities and partition the non-linear optimization problem into sub-problems. GAML was used to create a case study for 3D shoulder model to benchmark our approach with up to 5000 constraints.

**Results:**

Our results show that the computation time was reduced from exponential time to linear time and the error rate between the partitioned and non-partitioned approach decreases with the increasing number of constraints. For the largest constraint set (5000 constraints), speed up was over 2689-fold whereas error was computed as low as 2.2%.

**Conclusion:**

This study presents a novel approach to group anatomical constraints in 3D human shoulder model using community detection algorithms. A case study for 3D modeling for shoulder models developed for arthroscopic rotator cuff simulation was presented. Our results significantly reduced the computation time in conjunction with a decrease in error using constrained optimization by linear approximation, non-linear optimization solver.

## Background

This paper introduces a novel approach to speed up the exponential computation time in our optimization model for geometry constraint solving. In our previous work, we had introduced Generative Anatomy modeling Language (GAML) [1]. GAML helps generate variation of 3D virtual human anatomy in real-time. It also incorporates anatomical constraints to create anatomically correct variations of 3D models. Our goal of developing GAML was to minimize numerous iterations in the process for designing and modeling of 3D anatomically correct medical models. This modeling process necessitates involvement of expert physicians even for rudimentary geometry modifications. This protracts the model generation phase mostly due to the various challenges in collaboration of different parties such as i) hectic schedule of the physicians, ii) lack of tools to collaborate for people from various disciplines, iii) communication problem among engineers, designers, and physicians related to medical terminology, and iv) lack of anatomy knowledge of 3D designers, engineers etc. Besides, each design iteration causes manual alterations to ensure that the model is anatomically correct. Therefore, we proposed GAML (1) to avoid the lengthy consultation to expert opinion for basic anatomical verifications, (2) to eliminate manual work to speed up the design process, and (3) to increase the collaborative efforts between designers, engineers, and physicians.

GAML framework allows users to modify 3D models using human readable commands. These commands can be basic model modification commands (e.G. *affine* transformations, deformation) in conjunction with geometry constraints. Geometry constraints can be dynamically added to and removed from the 3D virtual model depending on the anatomy. The ultimate goal of GAML is to manipulate 3D models while satisfying any imposed geometric constraints of the anatomy. The original motivation of GAML stems from the modeling challenges embodied in complex human anatomy.

GAML has commands for modeling anatomy using constraints and joint connections. We defined “Joint” as an abstract definition where any type of geometry constraints and joint type are stored [1]. For instance, human anatomy consists of three main types of joints which total up to 360 joints. These joints types are Fibrous, Cartilaginous, and Synovial joints. Each of these joint types have distinct constraints; Fibrous joints are fixed and immovable, while cartilaginous joints are slightly moveable and Synovial joints are freely moveable. In GAML, these joints can be added in between each bone, muscle, vein, skin etc., which overall can be used to build a 3D model for complex anatomical structures or systems.

In GAML, we used Powell’s nonlinear derivative-free constrained and non-linear optimization solver; Constrained Optimization by Linear Approximation (COBYLA) [2, 3]. According to our benchmark results, the computation time for our optimization model exhibited non-linear computation time after number of joints exceed 30 joints (≈120 constraints). We noted that the computational time increases significantly (e.g. 5000 constraints with 4 h’ computation time). This eliminates the practical use of GAML (real-time performance) as each single modification command requires re-computation of the solution which takes a lot of time. In this study, we introduce a novel solution to overcome this computation time problem for increasing number of joints (constraints). We partition the optimization problem in to smaller sub-problems by introducing additional constraints for each sub-problem. This allows us to concurrently compute the solution for the sub-problems independent from each other while retaining the original problem. Especially, in large number of constraints, solving partitions (sub-problem) of a problem compared to solving an original non-partitioned problem eliminates the exponential execution time and results in linear computation time.

The literature for this work stretches over multiple research areas such as problem decomposition with Divide and Conquer or sub-space techniques applied for non-linear optimization, Quadratic Programming (QP), or Mixed Integer Non-linear Programming (MINLP). In general, the purpose is to partition the problem using context information of the problem (e.g. graph partitioning) or linearize the problem or transpose the problem to search algorithm to find the global optimum. Moreover, division of a problem into smaller sub-problems is used to reduce time during constraint solving. Throughout the paper, optimization problem and problem are used interchangeably.

### Divide and conquer approaches

Divide and conquer approach causes additional computations for each sub-problem due to its recursive nature. In [4], a backtracking algorithm is used to overcome these additional computations. All sub-problems are solved recursively via backtracking and solutions are then combined until global constraint is fulfilled. Redundant checks are eliminated to increase speed; this increases the search space complexity which forbids proposed solution to work on large problems. Freuder et al. [5] used decomposition to extract a sub-problem from constraint satisfaction problem. This approach dynamically eliminates failed or not feasible solutions with forward checking to speed up computation. Forward checking used in this study is a form of backtracking search, which can cause late detection of conflicts/failures.

There are several methods which can be used in large-scale bound-constrained optimization problems. One example is the active-set method which is used for a primal and dual formulation of a QP According to Forsgren et al. [6], the aim of the method is to estimate the optimal set of constraints that are satisfied with equality. Furthermore, a standard active-set method has two phases; first phase will be ignoring the objective function where a feasible point is found; the second phase is to minimize the objective function while keeping its feasibility. The time complexity of the active-set method for large-scale problems has the worst-case complexity of *3*^n^ [7]. Solving of *QP* is needed for Support Vector Machine (SVM) training [8]. A study in [8], introduced Sequential Minimal Optimization (SMO) for SVMs where in this case QP is divided into sub-problems to allow linear memory and run times. For non-linear cases, SMO had 15 times speed up compared to the chunking approach [9]. Hsieh et al. [10] used divide and conquer method to divide large sample data to be classified with SVM. Two-step kernel k-means [11] clustering was used to cluster the large scale data in to sub-problems to be classified. Sub-problems are solved separately and solutions from sub-problems are used to solve the original problem. Divide and Conquer-SVM achieved 96% accuracy with 100 times speed up compared to Library for Support Vector Machines (LIBSVM) [12]. During experimental results, 100 different parameter settings were tested. Out of 100 settings LIBSVM was faster than Conquer-SVM on only 4 settings.

### Sub-space approaches

Sub-space techniques are used to solve large non-linear optimization problems [13]. Reducing the computation cost and memory size is one of the advantages of the subspace techniques. According to Gould et al. [7], the problem structure is important considering the size of problems: partially separable problems are easier to handle than intense problems.

One of the common approaches for sub-space technique is based on the linearization of non-linear constraints using the Sequential Quadratic Programming (SQP). Byrd et al. [14] used an approach to solve sub-problems by SQP. In this approach, each sub-problem is solved with trust regions [15]. Trust regions are subsets in quadratic functions which are thought to be correct. Use of trust regions creates efficiency and robustness which allows the solving of large problems. Iterations to solve sub-problems slow down the overall execution time due to the use of lower order corrections and the use of tight accuracy for refining the solutions. In [16], a large-scale non-linear programming algorithm was introduced to solve the barrier problem, which is a continuous function, while the point approaches the boundary of the feasible region of a problem, the value on the point increases to infinity [17], with the use of trust regions. The algorithm is based on SQP and interior point methods. In this algorithm [16], the trust regions are used for second derivatives to solve the barrier problem. The idea behind the SQP is to solve non-linearly constrained problems by minimizing quadratic approximations to the Lagrangian function to the linearized constraints [18]. According to Tillman et al. [19], the Generalized Reduced Gradient (GRG) method [20], modified sequential simplex pattern search [21], and the generalized Lagrangian function method [22] are effective on large-scale non-linear programming problems. The GRG method derived from reduced gradient method by using non-linear constraints and bounds. The method uses a direction for independent variables and a direction is considered for dependent variables. Derivative is found by calculating the gradient of the objective function. However, there is no corroboration that one of these algorithms is the optimal in order to solve general non-linear programming problems [23]. Overall, SQP is an iterative method; thus, it requires more computation time than our proposed approach. Moreover, the SQP assumes that objective function and constraints are second order differentiable.

MINLP is another area, where problem partitioning can be used to improve the computation performance. In [24], a constraint-partitioning method, a partition-and-resolve procedure for solving P_t_ (CPOPT), was used to find local optimal solutions for large-scale MINLPs. In this approach violated global constraints are efficiently resolved using extended saddle points (decomposed sub-problems can be achieved from the original problem) [25] and large scale MINLPs are automatically partitioned to the optimal number of partitions to lower the search time. CPOPT was compared to a MINLP approach based on branch and bound [26] and Branch-and-Reduce Optimization Navigator (BARON) [27] using 22 large problem benchmarks from a collection of MINLP (MacMINLP) library [28]. In [26], a MINLP approach based on branch and bound tree search with SQP was used. In this approach branching is carried out after each iteration which allows the non-linear problem to be solved during searching of the tree, while BARON is a mixed integer constrained solver with branch and reduce. Out of 22 large problems MINLP approach based on branch and bound couldn’t find a feasible solution for 13 large problems while BARON couldn’t find a feasible solution in 12 large problems respectively in the time limit of 3600 s. For the large problems with feasible solutions CPOPT was only slower in 2 large problems compared to MINLP approach based on branch and bound and BARON. For the smaller problems, BARON and MINLP approach based on branch and bound had average solution times of 4.59 and 5.45 s, while CPOPT had an average solution time of 8.4 s due to partitioning overhead. Nagarajan et al. [29] introduced an adaptive and multivariate partitioning algorithm for solving MINLPs. Adaptive multivariate partitioning, partitions domains non-uniformly according to the best-known local solution. In this work, a user parameter is used to scale the partition’s size, thus affecting the number of partitions and the rate of convergence. The proposed approach performed better than BARON [27] in 26 out of 32 for MINLPLib [30] instances.

## Methods

### POM-GAML overview

**Fig. 1:**
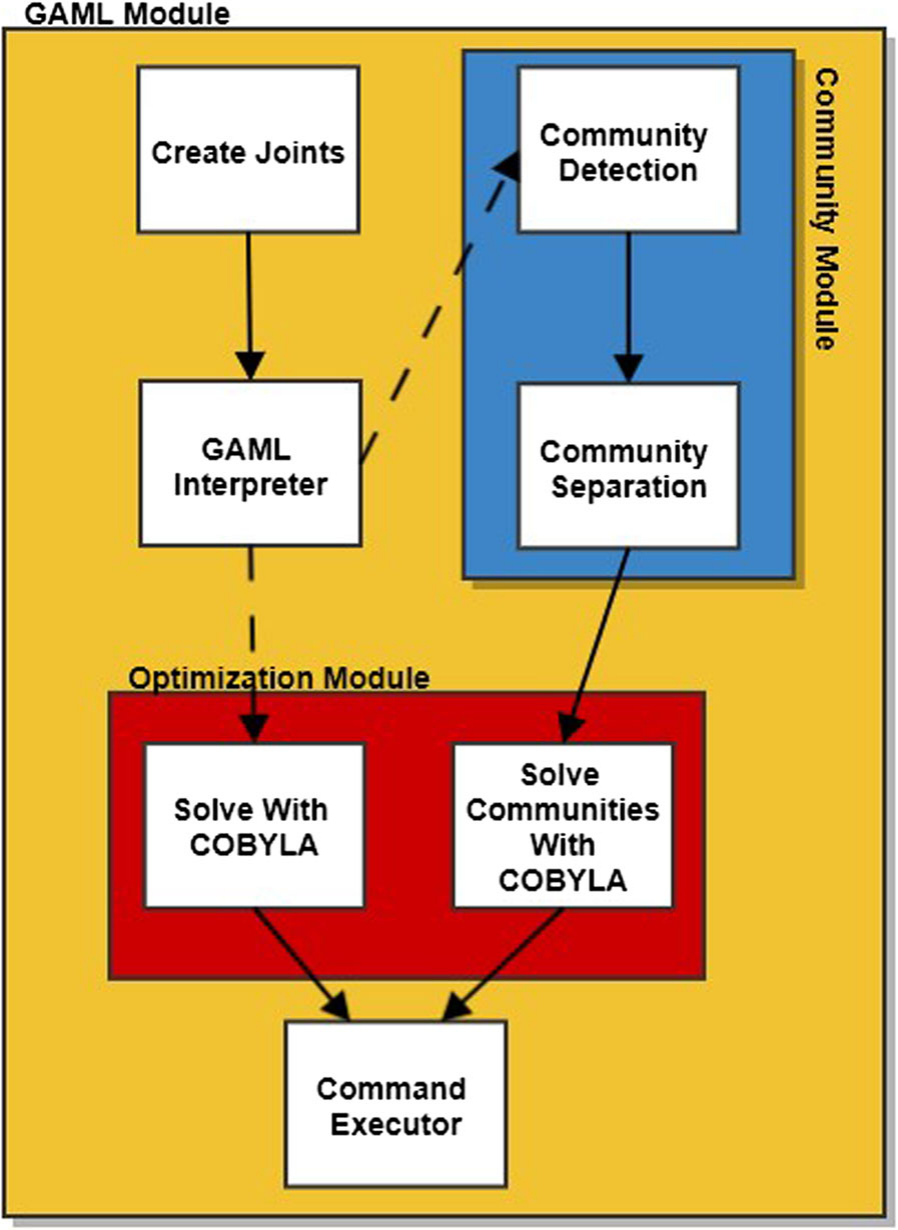
Framework Architecture. Solid lines show the order of operation, while dotted lines show decision operation

Our Partition-based Optimization Model for Generative Anatomy Modeling Language (POM-GAML) approach was developed as a module as an extension to our GAML framework. The current design has (a) GAML interpreter for command processing and constraint entry, (b) Community module, and (c) Optimization module. In the GAML, joints are created with user commands which then sent to GAML interpreter for model update. The solution to the optimization model can be performed either executing solver directly on the optimization model or partitioning the problem (community approach) and following solver execution. The decision is made based on the preference; if non-community approach is preferred, the command is sent to optimization module and executed. If the community approach is preferred, the request is sent to the community module first where the communities are detected, and the optimization problem is partitioned. The module revises the problem and redirects the execution to the optimization module for solution. In POM-GAML, a community or cluster is an abstract definition for group of nodes that are close enough and densely connected to each other. At present, the communities are detected based on the Clauset Newman Moore (*CNM*), Density-based spatial clustering of applications with noise (*DBSCAN*), and k-means clustering approaches. Architecture of our framework is shown in Fig. 1.

### Optimization model

Optimization model aims at computing the closest possible location (e.g. goal location) given a desired motion for each joint. In other words, our model seeks to minimize the error between the desired location and goal location. That being said, all joints that have designated motions by user are placed in objective function. Similarly, if there are no prescribed motion for a joint, it will be dropped from the objective function. The non-linear solver tries to minimize the overall error for all the joints in the objective function. Both joints and the constraints can be dynamically changed/updated in the optimization model prior to prior to each non-linear solver step.

Our previous optimization model [1] can be seen in Table 1. In our model, *p*_*i*_ is a 3D position of a joint (*J*_*i*_), where *J*_*i*_ can be an arbitrary node in or a node attached to a 3D Mesh (*M)*. Number of joints (*N*), can be dynamically modified by adding and removing joints. In between each joint couple *i (p*_*i*_*)* and *j (p*_*j*_*),* there could be up to four different types of constraints (Eqs. 1–4 in Table 1) to constrain the movement. In our model, unique constraint sets are formed for each constraint type. For instance, Eq. 1 is defined for the absolute distance constraint and set *A* indicates the set of absolute distance constraints. Similarly, Eq. 2, Eqs. 3–4 are for the angle constraints and the flexibility constraints and set B and C are designated for the set of angle and flexibility constraints respectively. *Dist*_*ij*_ is the original distance while *∆d*_*max*_ is the maximum displacement allowed between the joint couple *p*_*i*_ and *p*_*j*_ using the stiffness ratio *k*_*l*_. *p*_*io*_ is the initial point for joint *p*_*i*_ and *θ*_*ij*_ is the maximum angle that joint *p*_*i*_ is allowed to pivot about joint *p*_*j*_*.* (*p*_*io*_ − *p*_*j*_)_*axis*_ and (*p*_*i*_ − *p*_*j*_)_*axis*_ are the directional vectors in the x, y or z axis’ to accommodate any lock along the axis of rotation (Eq. 2a). For the angle constraint, *p*_*io*_ is the original position of *p*_*i*_ and is the direction vector of *p*_*io*_ and pivot point of *p*_*j*_ is (*p*_*io*_ − *p*_*j*_). The direction vector between *p*_*i*_ and pivot point of *p*_*j*_ is (*p*_*i*_ − *p*_*j*_). Further details of the optimization model and use cases can be found in our previous work [1].

**Table 1:** Optimization model [1]

*Arg Min:* $\sum \limits_{l=1}^N{k}_l\left|{p}_l-{p}_{Destination}\right|$		
*Subject to:*		
*Dist*_*ij*_ − |*p*_*i*_ − *p*_*j*_| = 0	for (*i,j*)∈ *A*	*(1)*
${\mathit{\cos}}^{-1}\left(\frac{\left({p}_{io}-{p}_j\right)\bullet \left({p}_i-{p}_j\right)}{\left\Vert \left({p}_{io}-{p}_j\right)\right\Vert \times \left\Vert \left({p}_i-{p}_j\right)\right\Vert}\right)-{\theta}_{ij}<0$	for (*i,j*)∈ *B*	*(2)*
(*p*_*io*_ − *p*_*j*_)_*axis*_ − (*p*_*i*_ − *p*_*j*_)_*axis*_ = 0		*(2a)*
*Dist*_*ij*_ − *∆d*_*max*_ − |*p*_*i*_ − *p*_*j*_| < 0	for (*i,j*)∈ *C*	*(3)*
|*p*_*i*_ − *p*_*j*_| − *Dist*_*ij*_ − *∆d*_*max*_ < 0		*(4)*
*i*, *j* ∈ *J and i*, *j* ⊆ *M*, *k*_*l*_ > 0, *A* ∩ *C* = ∅		

### Partition based constraint model

**Fig. 2:**
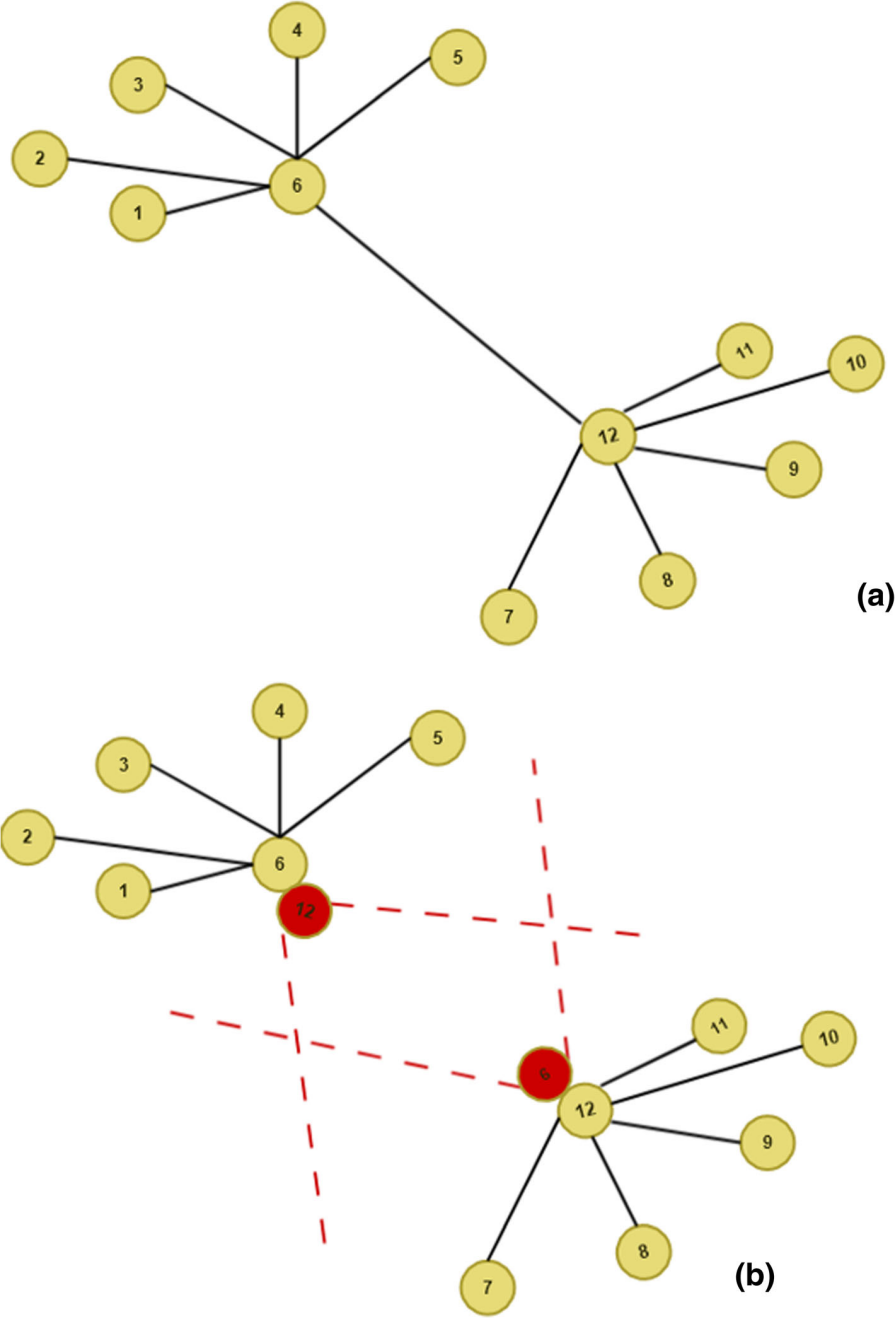
(**a**) Connected Joints, (**b**) Partition of joints (red joints are virtual)

In order to partition the model (as seen in Fig. 2), we first determine the candidate nodes where a split of the model can occur. These candidate nodes are possible centers of the communities that are determined with community detection algorithms using joint graphs (see Section 4). The split starts with geometry-based operation in the constraint hierarchy. The result of the operation creates additional optimization models with the number of splits. For instance, one split creates two separate geometry and optimization models. The split operation is performed on a pair of nodes with duplicating these candidate nodes. The next operation is to remove all the constraints amongst the nodes of the splits from each other starting with pairs. A duplicate node will serve as a virtual node that represents the node of the split pair that remain in the other split. This is to ensure to retain the original joint hierarchy at each split. The key step here is that we introduced a new constraint for these candidate pairs. Unlike the original problem, this will introduce a flexible constraint that anticipates the location of the virtual node (e.g. the other attached node) within an estimated range of motion. We determined this predication with a cone like motion (as seen in eq. 9) with a virtual joint (*v)*.

In our model (as seen in Table 2), *V* is the set of virtual joints. Virtual joint holds information about the disconnected joints and enforces its constraints. *v*_*i*_ is the virtual joint of joint *p*_*i*_ and *v*_*j*_ is the virtual joint of joint *p*_*j*_. *P*_*t*_ is the partition, and none of the joints in one partition can be shared or exist in another partition, unless it is a virtual joint. This imposes the splits are completely disjoint model and share no nodes or joints. In Equation 9, *r* is the radius of the cone, while *a*_*ij*_ is the angle. Angle of cone is a preset to 30°, which is used to create the anticipated range of motion of virtual nodes. We empirically observed that the change of preset angle makes no significant difference in the constraint sets over 50. Radius of the cone is calculated in Equation 10 and *D* is the set of cone constraints.

**Table 2:** Optimization Model for Partitions

*Arg Min:* $P{\left(\sum \limits_{l=1}^N{k}_l\left|{p}_l-{p}_{Destination}\right|\right)}_t$		
*Subject to:*		
*Dist*_*ij*_ − |*p*_*i*_ − *p*_*j*_| = 0	for (*i,j*)∈*A*_*t*_	*(5)*
${\mathit{\cos}}^{-1}\left(\frac{\left({p}_{io}-{p}_j\right)\bullet \left({p}_i-{p}_j\right)}{\left\Vert \left({p}_{io}-{p}_j\right)\right\Vert \times \left\Vert \left({p}_i-{p}_j\right)\right\Vert}\right)-{\theta}_{ij}<0$	for (*i,j*)∈*B*_*t*_	*(6)*
(*p*_*io*_ − *p*_*j*_)_*axis*_ − (*p*_*i*_ − *p*_*j*_)_*axis*_ = 0		*(6a)*
*Dist*_*ij*_ − *∆d*_*max*_ − |*p*_*i*_ − *p*_*j*_| < 0	for (*i,j*)∈*C*_*t*_	*(7)*
|*p*_*i*_ − *p*_*j*_| − *Dist*_*ij*_ − *∆d*_*max*_ < 0		*(8)*
$2\operatorname{sgn}\left({p}_i{p}_j.{p}_i{v}_j\right){\times \mathit{\tan}}^{-1}\left(\frac{r}{\left\Vert {v}_j\right\Vert}\right)-{a}_{ij}<0$	for (*i,j*)∈*D*_*t*_	*(9)*
$\left(\frac{\tan \left({a}_{ij}\right)}{\left\Vert {v}_j\right\Vert}\right)<0$		*(10)*
|*p*_*i*_ − *v*_*j*_| < 0		*(11a)*
|*p*_*i*_ − *v*_*i*_| = 0		*(11b)*
*i*, *j* ∈ *J and i*, *j* ⊆ *M*, *k*_*l*_ > 0, *A* ∩ *C* = ∅ , *P*(*A*)*m* ∩ *P*(*A*)*n* = ∅ , *v*_*i*_ = *p*_*i*_, *v*_*j*_ = *p*_*j*_, *v* ∈ *V*, *v* ∉ *A*, *B*, *C*		

The constraint among the candidate pairs is generalized with a distance constraint. This distance constraint is formulated as (Equation 12);

12 $$ C(p)=\left\Vert \left(p-{p}_o\right)-r\right\Vert =0 $$

In equation 12, *r* is the current distance, *p* and *p*_*o*_ are joint current and initial locations respectively. In the context of our model, *p* and *p*_*o*_can be node and virtual locations in turn. In order to get rid of the L1 norm, we could redefine the equation in L2 norm as (Equation 13) to eliminate the square root in the formulation;

13 $$ C=C(p)=\frac{1}{2}\left[{\left(p-{p}_o\right)}^2-{r}^2\right] $$

At all times, the both virtual nodes should retain the initial distance to ensure the integrity of the original problem. The derivate of the constraint with respect to solver iterations of the model should also satisfy the constraint equation. This constraint is defined with chain rule as the following (Equation 14);

14 $$ \dot{C}=\frac{dC}{di}=\frac{\partial C}{\partial p}\frac{\partial p}{\partial i}=\left(p-{p}_0\right).\frac{\partial p}{\partial i}=\left(p-{p}_0\right).\dot{p} $$

Similarly, the second derivative should also satisfy the imposed constraint (Equation 15);

15 $$ \ddot{C}=\frac{d\dot{C}}{di}=\frac{d\Big(\left(p-{p}_0\right).\dot{p\Big)}\ }{di}=\frac{\partial C}{\partial p}\frac{\partial p}{\partial i}={\dot{p}}^2+\left(p-{p}_0\right)\ \ddot{p} $$

All the constraints equations above should satisfy the following per iteration (Equation 16);

16 $$ C=\dot{C}=\ddot{C}=0 $$

Therefore, we could predict that the (*p* − *p*_0_) vector will be orthogonal to the $\dot{p}$. We could simply represent the $\dot{p}$ with λ*n*, which denotes that the change in *p* should be in the direction of the unit vector *n* with some λ amount. The vectors *n* and *p* − *p*_0_ which is *r* (e.g. from the equation of *C* (Equation 16)) should be orthogonal. Therefore, we incorporated this orthogonality constraint within a cone that sweeps the possible range of motion of *p* described in the revised model in the previous section. We could also extend the formulation for the derivation of the relation between λ and *r* with using second derivative of constraint equation (Equation 17);

17 $$ \ddot{C}=0={\uplambda}^2n.n+\left(p-{p}_o\right)\ddot{p} $$

This reduces to the following relation (Equation 18);

18 $$ \mid \uplambda \mid =\sqrt{-r.\ddot{p}} $$

### Community detection in joint graphs

A hierarchical graph data structure allowed us to extract the connectivity among joints and their organization [31]. The graph can be utilized to find communities of joints for possible splits. We propose to determine these joints with community/cluster detection algorithms/approaches. In our joint hierarchy, connectivity between joints also represent the constraints of joints. We hypothesize that joint communities with more constraint density (e.g. number of constraints per node) could be good candidates for optimization model partition. Therefore, we seek to find the cluster of joints with highest density (density of features in the context of data mining) which is the main goal of clustering/community detection [32]. However, there is no single solution for clustering/community detection [33]. In this work, we used three different community detection algorithms; *CNM* [34], *DBSCAN* [35], and *k-means* clustering [36]. The community detection algorithms inherently take the links (e.g. constraints) into account whereas in the clustering algorithms we used the constraints for distance computations. In the manuscript, although there is a slight difference, clustering and community detection phrases are used interchangeably.

In a common graph structure, vertices are used for nodes and edges are used for hierarchical links. In our framework, a joint is used as nodes and the constraint represents an edge to form a graph structure. Once the graph structure is generated, communities in the graph are identified. The parameters (ε, MinPts, numberofClusters, etc.) of the selected community detection algorithm is based on the prescribed input. Once the communities are identified, joints in the graph are partitioned to their communities. The process is basically performed in two steps; (1) the connection between the joint pairs which belong to separate communities is deleted, (2) a virtual joint for each joint is inserted into each community that would represent the deleted joint.

**Fig. 3:**
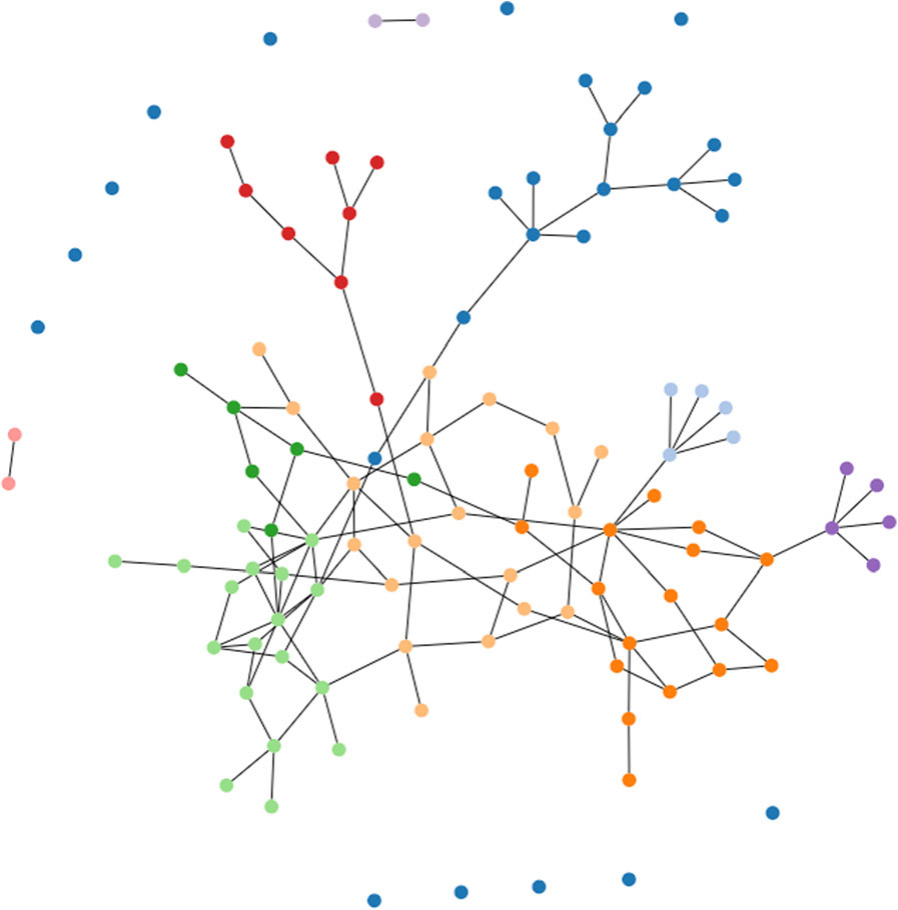
CNM for 5000 constraints

In *CNM*, modularity [37] is to measure the division strength of a network in to communities. Modularity is calculated as the ratio of number of edges in each community against the number if edges between the communities. CNM starts with the calculation of modularity at the node level. Each node is assumed as a community then the modularity between each pair of communities is calculated. Pair of communities with the highest modularity are combined together and this approach is carried out recursively. Modularity greater than 0.3 indicates a significant community [34]. All our constraint sets had a final modularity of greater than 0.3. *CNM* is an agglomerative hierarchical method and is based on greedy optimization. Time complexity of *CNM* is *O*(*V* ∗ *logV*) [34], where *V* is the number of vertices (joints) in the graph. Figure 3 visualizes *CNM* for the sample constraint set of 5000 in our test data.

Another approach used to find communities is *DBSCAN* with time complexity of *O*(*V*^2^). *DBSCAN* discovers high dense areas and detaches them from low dense areas. *DBSCAN* algorithm parameters are; the amount of joints required for a dense area (*MINPTS*) within the radius (ε) and a distance function of connectivity of joints in the graph. DBSCAN starts with finding the neighbors of every node, if two nodes are within the specified ε they are considered neighbors. DBSCAN groups neighbor nodes according to *MINPTS* and a distance metric. A high-dense area must have at least *MINPTS* within the distance metric. Unless the minimum number of points is satisfied the area is considered low-dense.

**Fig. 4:**
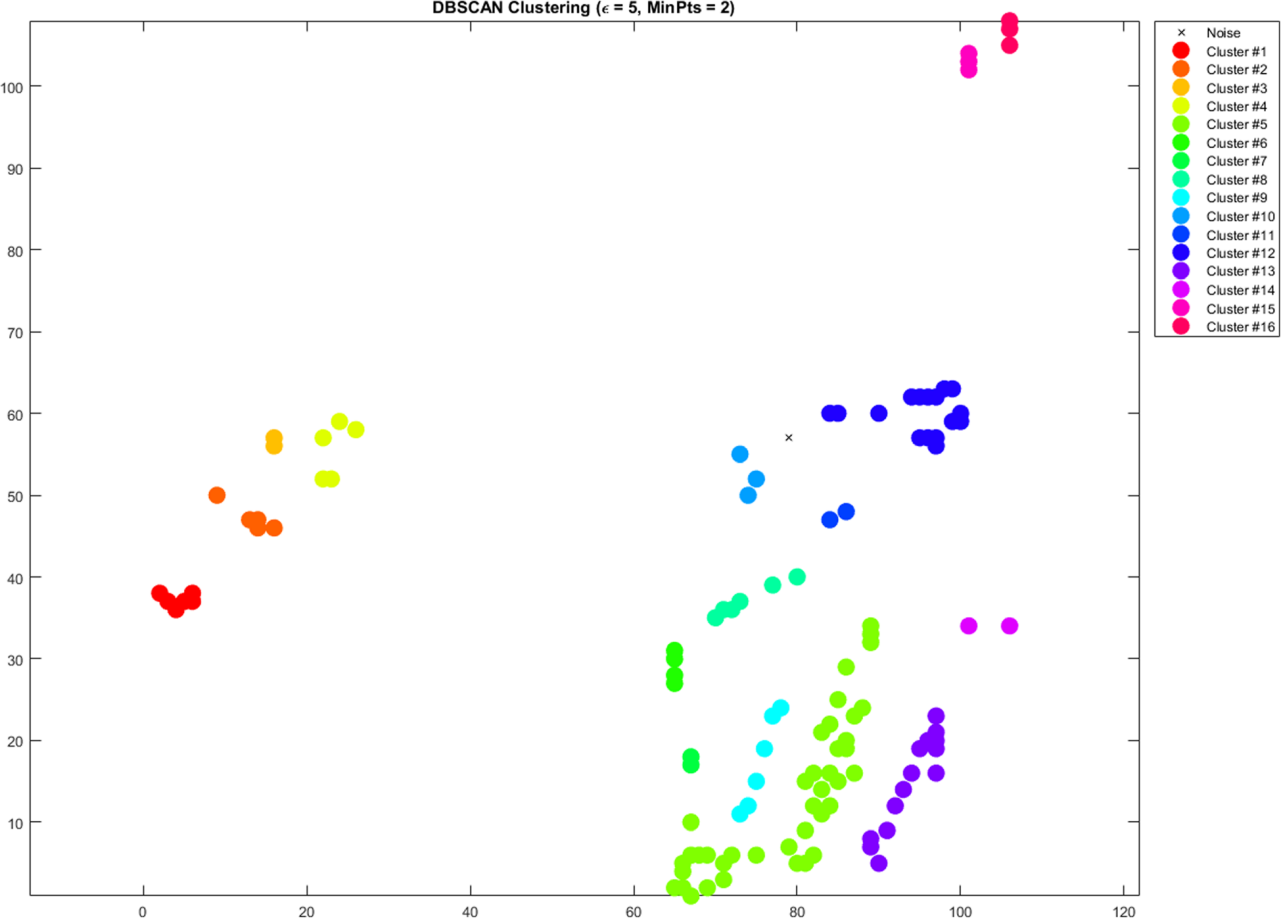
DBSCAN clustering for ε = 5 and MINPTS = 2 for the constraint set of 5000

**Fig. 5:**
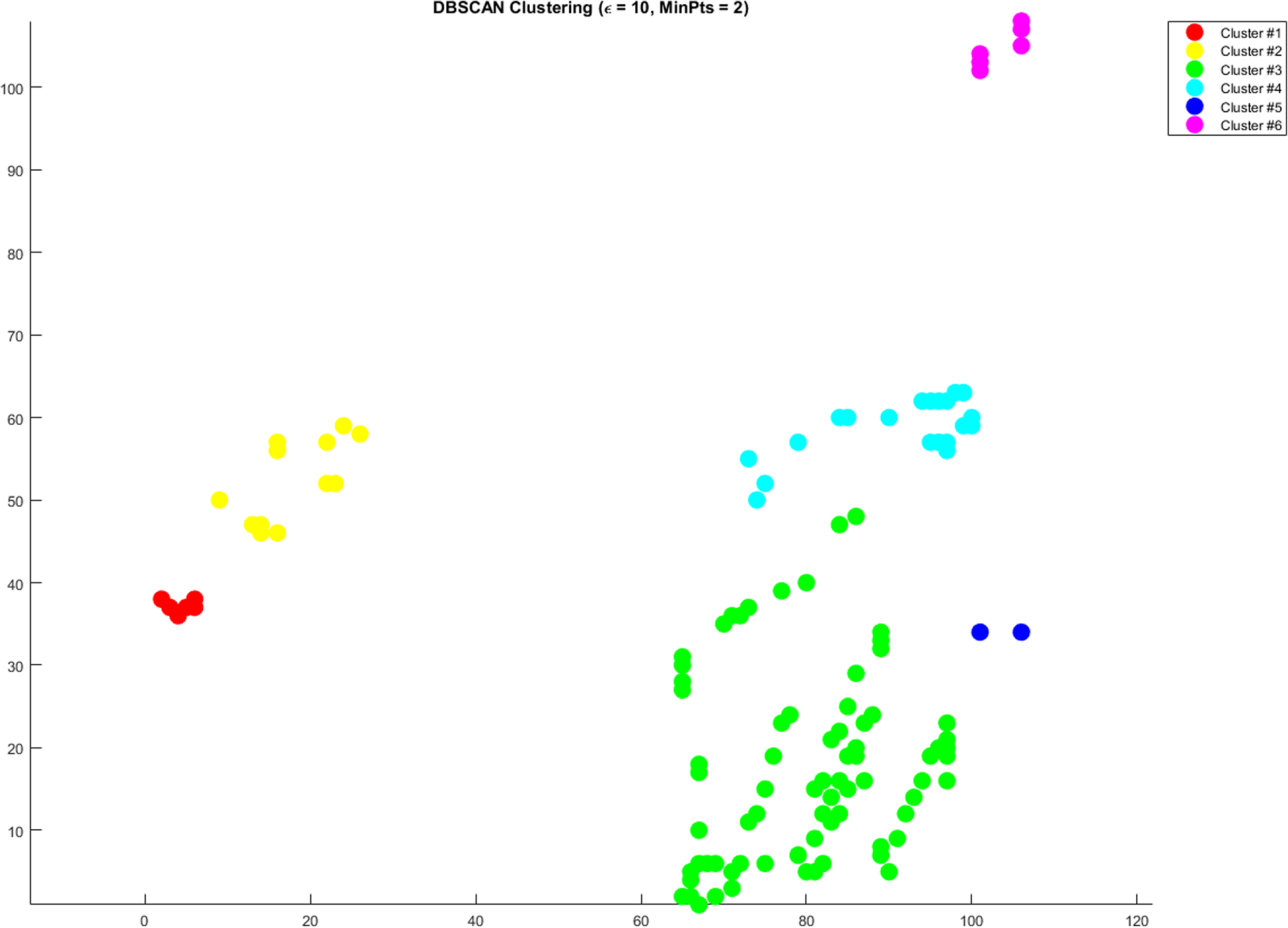
DBSCAN clustering for ε = 10 and MINPTS = 2 for the constraint set of 5000

For *MINPTS*, we selected 2 and for ε we used 5 (*DBSCAN5*) and 10 (*DBSCAN10*) respectively. Figure 4 shows the *DBSCAN* clustering for ε = 5 and *MINPTS*=2 for the constraint set of 5000, while Fig. 5 shows the *DBSCAN* clustering for ε = 10 and *MINPTS*=2 for the constraint set of 5000 of the same model data as used in CNM testing.

**Fig. 6:**
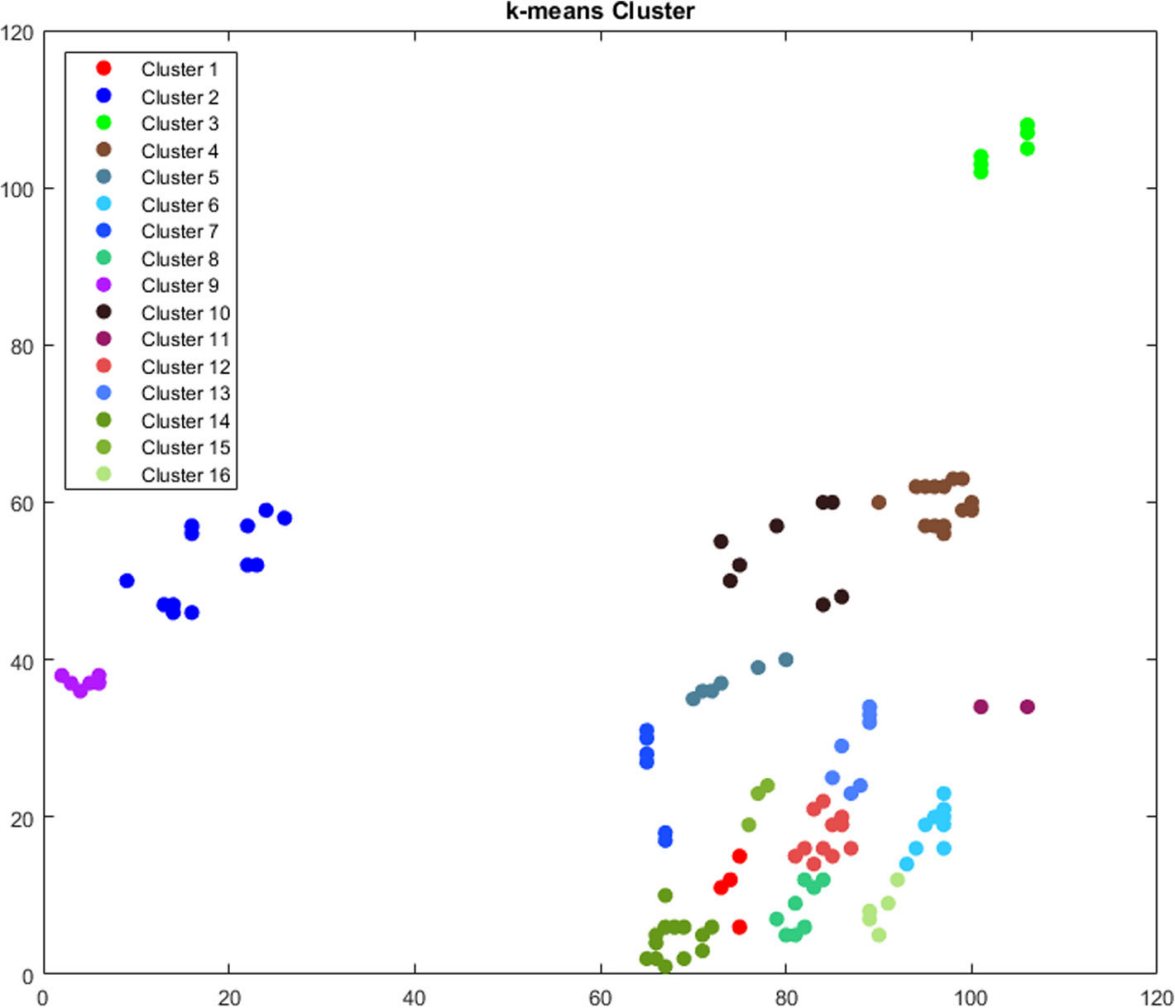
k-means Cluster for DBSCAN-5

**Fig. 7:**
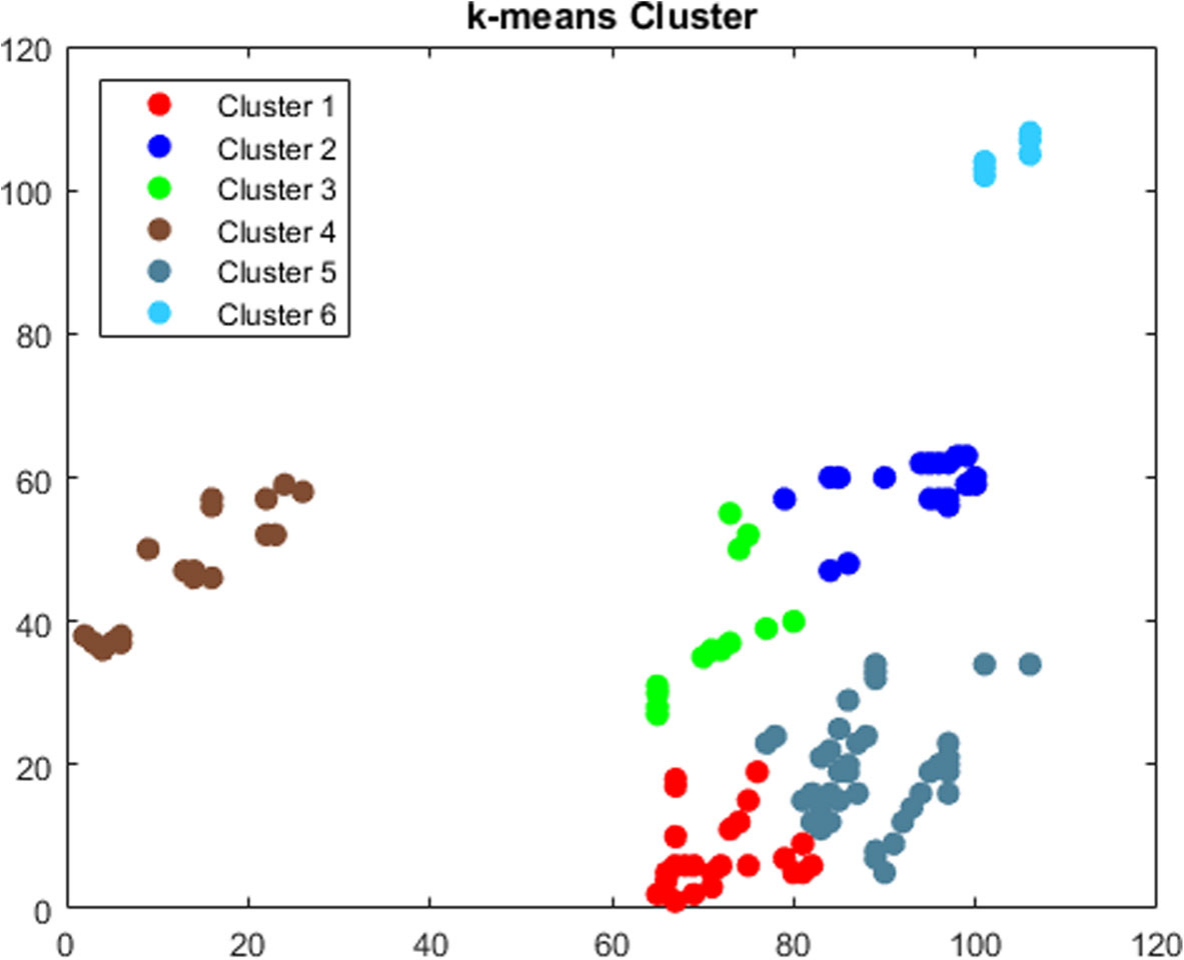
k-means Cluster for DBSCAN-10

The third approach used to find communities and partition an *N*-dimensional data into *k* sets is *k-means* clustering algorithm. One of the applications of the *k-means* is approximating a general distribution that estimates the distribution between the sample points [36]. A minimum distance partition of the sample points can be found by using *k-*means algorithm. *K-means* clustering in high dimension is a NP-hard problem and there are several partitioning algorithms called “center-based clustering” to improve quality [33]. The time complexity of the *k-means* algorithm in *d*-dimension is *O*(*n*^*kd*^). However, Hamerly et al. [38] stated that the worst-case time complexity of *k-means* is super polynomial. Finding the minimized sum of squared deviations of the partitions of the *k* sets is the goal of *k-means* clustering algorithm [39]. For our case, *k* was set to the same amount of communities as *DBSCAN* clusters. Thus, *k-means-5* and *k-means-10* have the same amount of communities as *DBSCAN-5* and *DBSCAN-10* respectively. Figures 6 and 7 show the k-means clusters for *DBSCAN-5* and *DBSCAN-10* respectively.

## Results

### Time and error

**Fig. 8:**
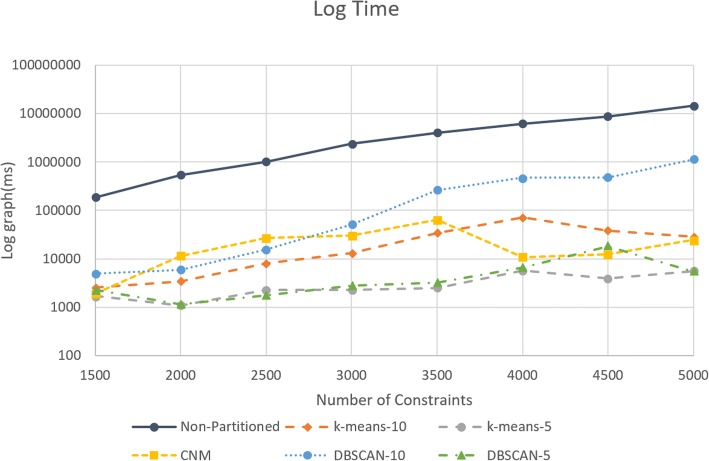
Log graph for execution time for increasing number of constraints

In order to measure the execution time and error, we generated random joints sets starting from 1500 to 5000 and used community/cluster detection algorithms used to partition the optimization model. For each constraint set, we performed the computation five times for each partitioning algorithm, and the average computation was used as a result. For performance measurement, an Intel Core i7-5820 K CPU with 16GB Ram and a GeForce GTX 970 GPU with Driver version 388.13 was used. We benchmarked the execution time (in log graph) for non - partitioned model and each of our differently partitioned communities as seen in Fig. 8. *COBYLA* was used for optimization model solver.

**Fig. 9:**
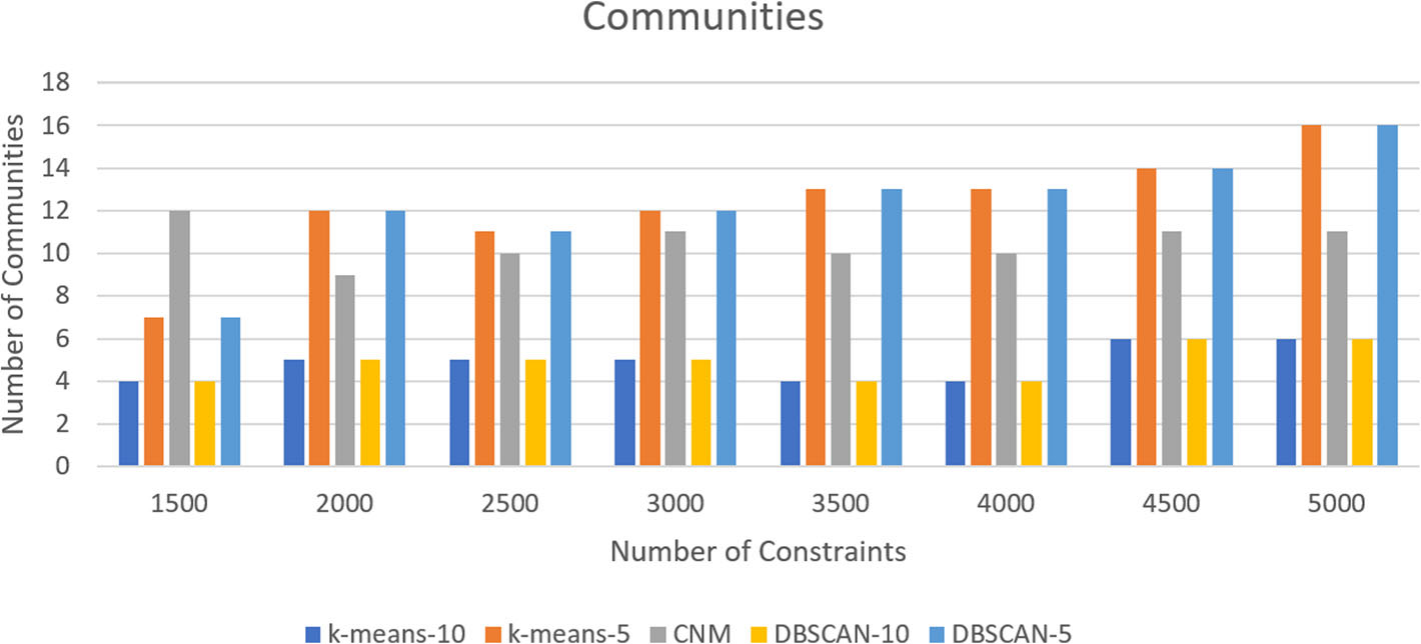
Number of communities for each constraint set and community detection algorithm

According to our results, execution times in partitioned base models achieve almost linear execution times with increasing number of constraints, whereas non-partitioned model demonstrate non-linear execution times as seen in log graph in Fig. 8. Regarding the execution time for partitioned communities, *DBSCAN-10* clustering execution time was higher in all constraint sets except for 2000 and 2500. In those sets of constraints, the difference in number of communities between *DBSCAN-10* and *CNM* were minimum; *DBSCAN-10* had 5 communities for both, while *CNM* had 9 and 10 communities respectively. Furthermore, for constraint sets for 2000 and 2500 *CNM* execution times have peaked (11,343 ms and 27,363.7 ms respectively), while *DBSCAN-10* execution times have dipped (5870.11 ms and 15,583.34 ms respectively). For the constraint set of 1500, *CNM* had the most number of communities (12 >communities), while above the constraint set of 1500 *DBSCAN-5* and *k-means-5* had the most communities. In all constraint sets, *DBSCAN-10* and *k-means-10* had the least amount of communities. *K-means-5* or *DBSCAN-5* had the fastest times for every constraint set out of each partitioned community detection algorithms except for the constraint sets of 2500 and 5000 where *k-means-5* was faster than *DBSCAN-5*. Figure 9 shows the number of communities for each constraint set and community detection algorithm. Table 3 shows the speed-up times for each partitioned algorithm. The maximum overall speed-up is achieved with *DBSCAN-5* and *k-means-5* with 5000 constraints with the execution time 2689.72 and 2677.52 compared to non-partitioned problem.

**Table 3:** : Speed-up(*x*-times) for each partitioned algorithm compared to non-partitioned COBYLA

# of Cons affected	k-means-10	k-means-5	CNM	DBSCAN-10	DBSCAN-5
1500	73.17	108.64	97.18	38.15	82.11
2000	159.55	498.42	47.57	91.92	474.40
2500	123.63	449.27	36.99	64.96	567.79
3000	181.20	1036.72	78.81	46.24	849.14
3500	118.93	1610.41	62.89	15.32	1248.50
4000	86.77	1068.89	578.53	12.95	927.99
4500	231.31	2218.13	701.81	18.38	477.99
5000	510.19	2677.52	601.19	13.18	2689.72

Partitioning the original problem in to sub-problems will introduce errors with respect to original problem. Error calculation is shown in Equation 19.

19 $$ \% error= Avg\left({\left|\frac{Partition- Non- Partition}{Non- Partition}\right|}_i\right)\times 100 $$

**Fig. 10:**
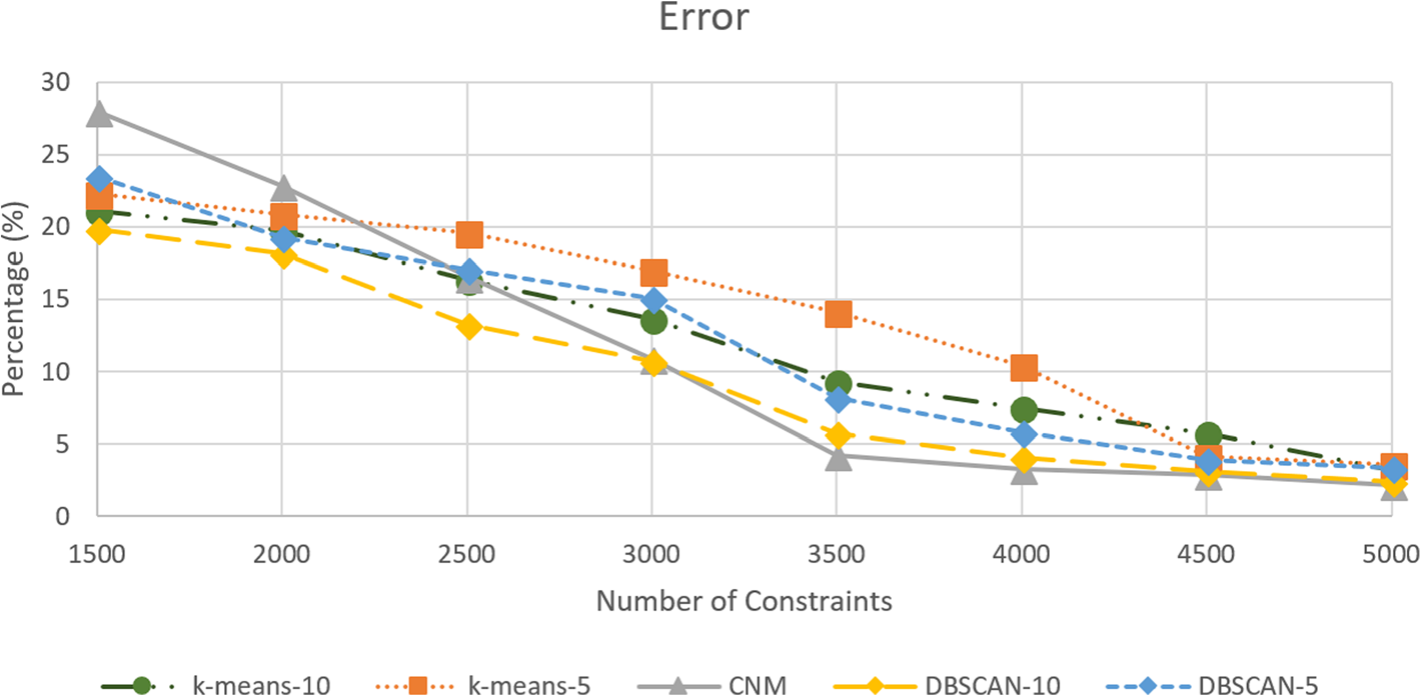
Error percentage for each community detection algorithm

**Fig. 11:**
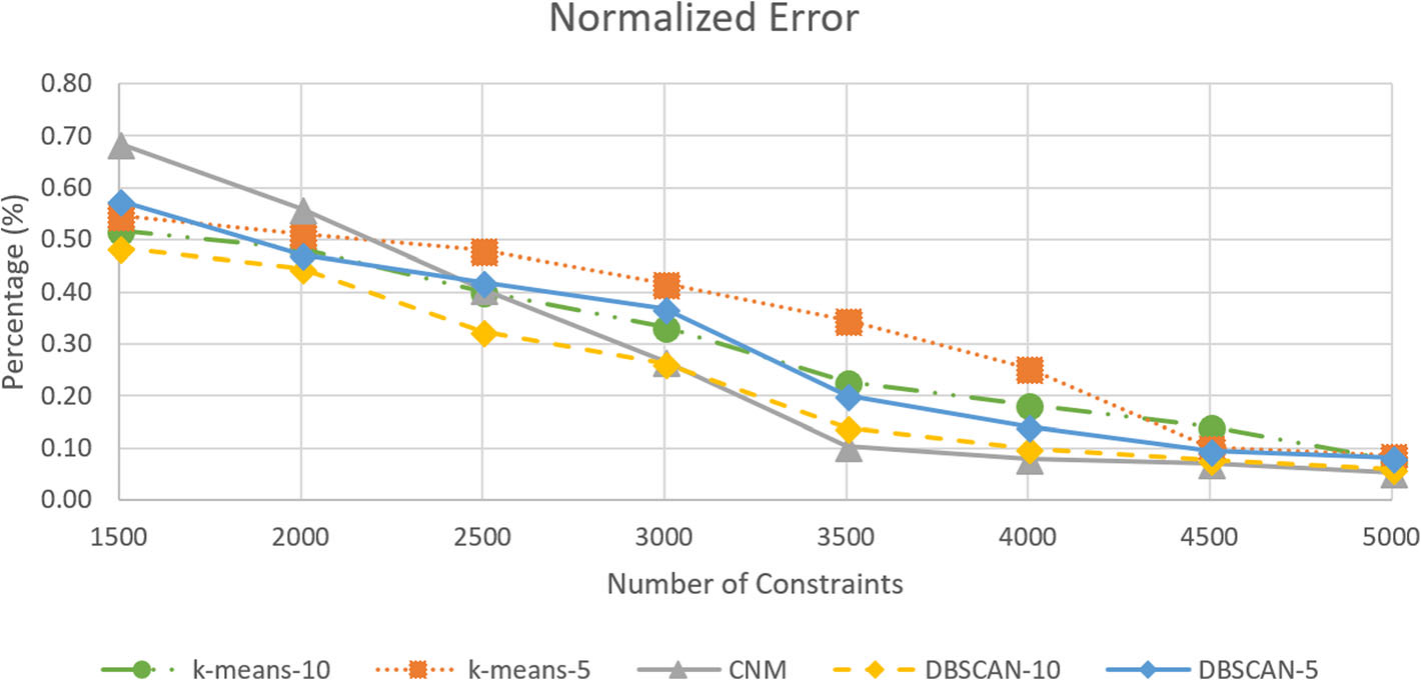
Normalized error percentage to the scene

We noted that errors for all the community detection algorithms are between 19.8 and 28% for 1500 constraints. For constraint set of 1500, *CNM* had the highest error percentage at 28.04%, while *DBSCAN-10* had the lowest at 19.84%. Between constraint set of 3000 and 3500, errors had the biggest decrease for *CNM* (10.89 to 4.26%)*, k-means-10* (13.63 to 9.32%)*, DBSCAN-10* (10.75 to 5.72%) and *DBSCAN-5* (15.09 to 8.2%). *K-means-5* had the biggest decrease in error between the constraint sets 4000 and 4500 by decreasing from 10.40 to 4.15%. At the constraint set of 3500, all partitioning algorithms reached below 10% error percentage except for *k-means-5* which stayed at 14.13%. For the constraint set of 5000, *CNM* reached the lowest error percentage at 2.2%, while *k-means-5* had the highest error percentage at 3.57%. The error graph can be seen at Fig. 10. Starting at the constraint set of 3500, *CNM* had the lowest error percentage compared to other community detection algorithms. Below the constraint set of 3500, *DBSCAN-10* had the lowest error percentage. Figure 11 shows the error percentage normalized to the scene (calculation can be seen in Equation 20). The scene magnitude was 40.86.

We computed our results with respect to normalized error. The error expression was given as in Equation-20. This stems from the empirical analysis that the errors are not noticeable to the eye for a model. The solution difference between the non-partition and original problem becomes minimal. Therefore, we consider the scene size or magnitude of the geometry. The error becomes less than 1% even for 1500 constraints as seen in Fig. 11.

20 $$ \% Normerror= Avg\left({\left|\frac{\frac{Partition- Non- Partition}{Non- Partition}}{SceneMag}\right|}_i\right)\times 100 $$

## Discussion

We experimented our optimization model for modeling human shoulder anatomy and human airway anatomy. The anatomies and its variations are important for simulation especially in training for difficulty scenarios.

### Shoulder anatomy

**Fig. 12:**
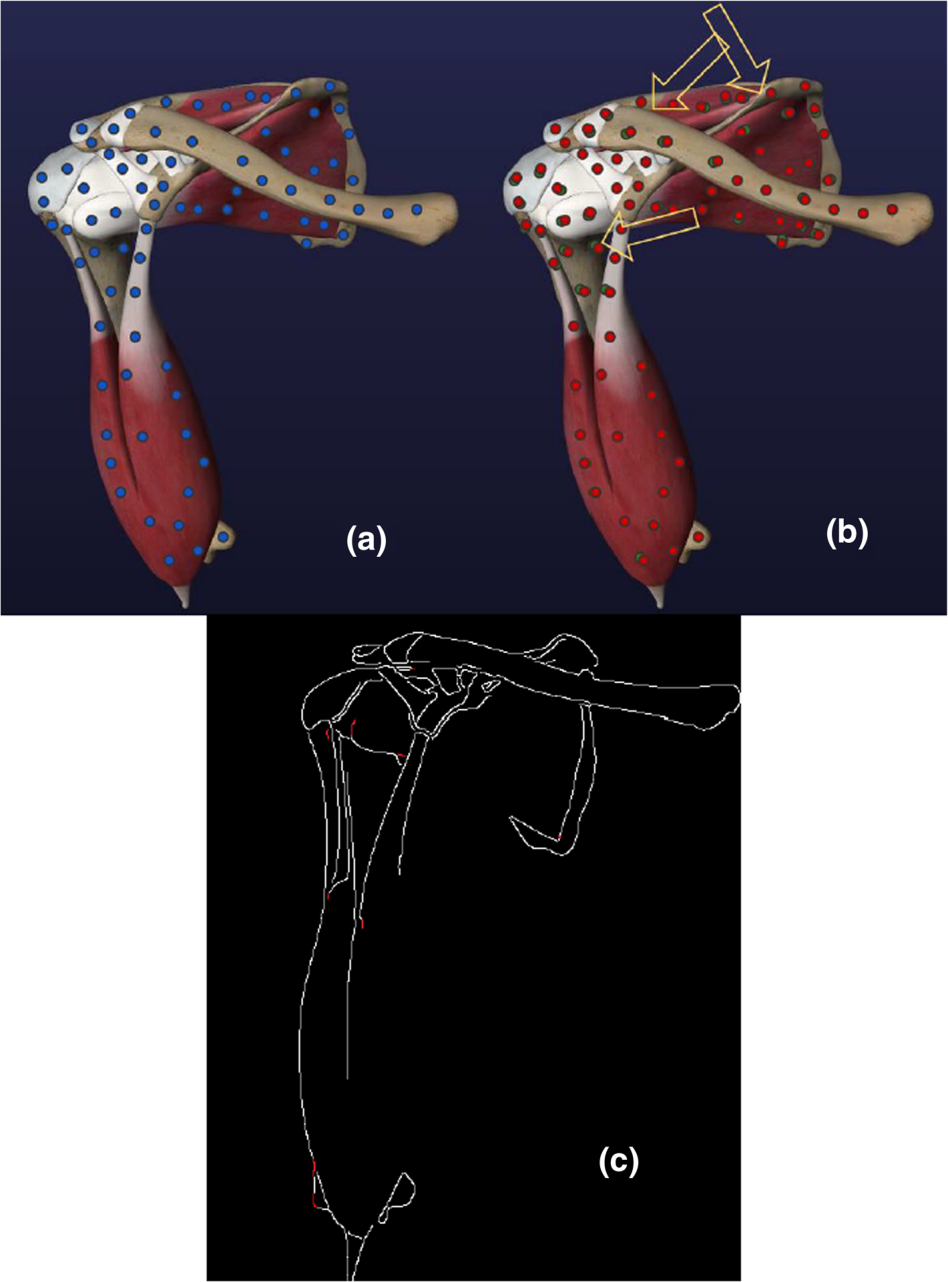
(**a**) Rotator Cuff Scene joints after solving with non- partitioned COBYLA and Partition Model, (**b**) Rotator Cuff Scene joints after solving with partitioned COBYLA and Partition Model, (**c**) Scene error comparison using Canny edge detection

The shoulder anatomy was developed for our arthroscopy surgery simulation for rotator cuff procedures. Arthroscopic rotator cuff surgery is a common minimally invasive shoulder surgery done through a scalpel incision for diagnosis and treatment of tissues and joints [40]. Rotator Cuff muscles attach shoulder blade to the upper arm and allow rotational motion of the shoulder [41]. We created a virtual 3D scene of the rotator cuff muscles and attached joints corresponding to constraint set of 5000. In the scene 25 3D models pertaining to shoulder anatomy with total of 160,392 vertices were used. We executed movement for Clavicle, Rotator Cuff muscles (Subscapularis, Supraspinatus, Infraspinatus and Teres Minor) and Humerus models. Figure 12a shows the 3D scene with joints which are represented as blue circles. Figure 12b shows the 3D scene with joints after motion using the partition and non-partition optimization model. In these figures, arrows indicate the transformation motion, red circles represent joints after solving with non-partitioned model using COBYLA solver while green circles represent the joints after solving with partitioned model using COBYLA solver. Visual errors for partitioned and non-partitioned COBYLA were not visible to the eye so we used Canny edge detection algorithm [42] in MATLAB to highlight the errors in the scene (Fig. 12c) In Fig. 12c the differences were marked with red.

### Airway anatomy

**Fig. 13:**
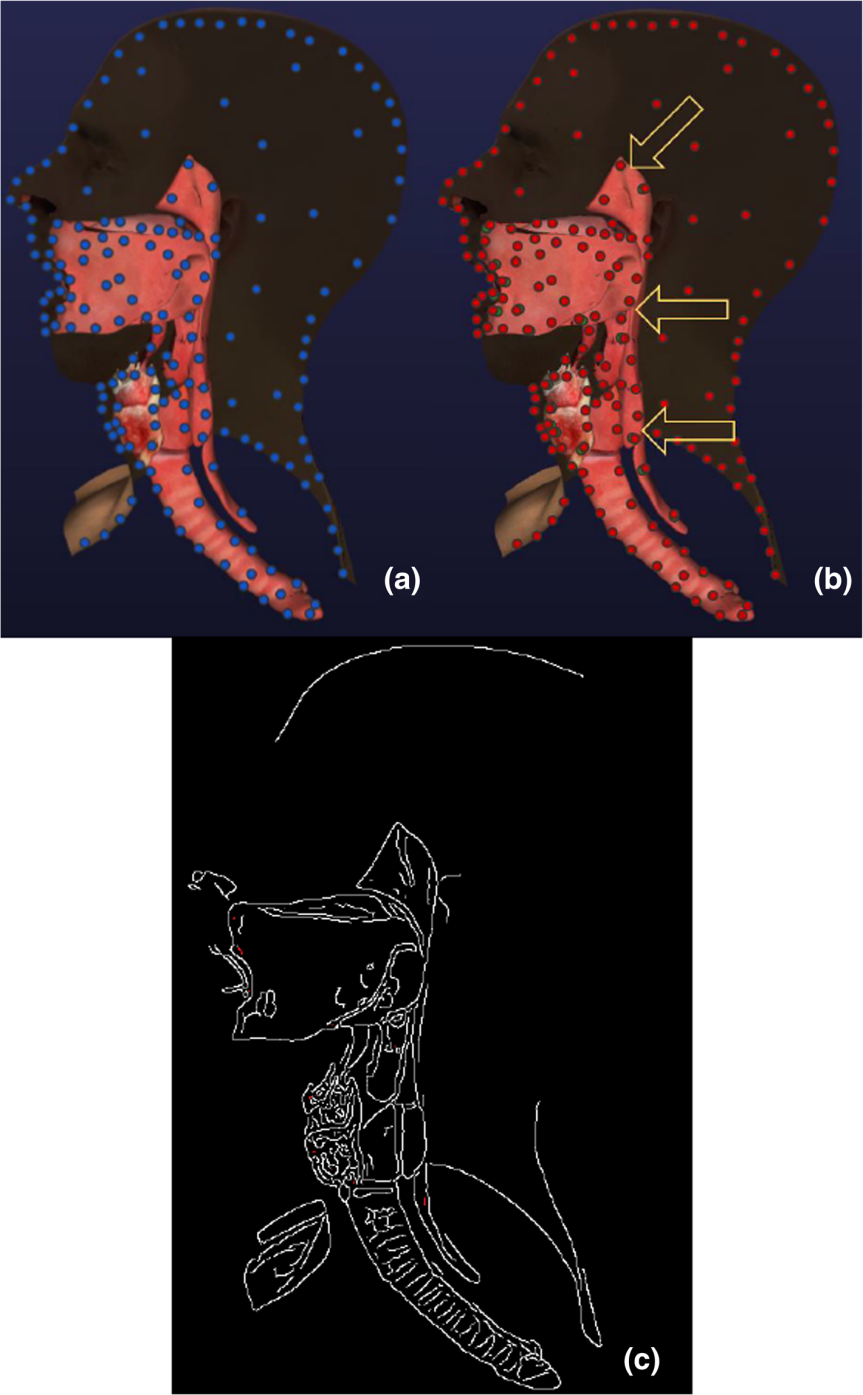
(**a**) Airway Scene joints after solving with non- partitioned COBYLA and Partition Model, (**b**) Airway Scene joints after solving with partitioned COBYLA and Partition Model, (**c**) Scene error comparison using Canny edge detection

Securing the airway on severely injured patients or a patient before a surgery is critical [43]. Life threatening complications can be avoided by securing the airway under 1 min. Otherwise, complications can cause permanent injuries or death. We created a virtual 3D scene of the airway anatomy and attached joints corresponding to constraint set of 5000. In the scene 8 3D models pertaining to shoulder anatomy with total of 214,686 vertices were used. We executed movement for larynx, thyroid cartilage, thyroid membrane, hyoid, cricoid membrane, cricoid cartilage, and tongue models. Figure 13a shows the 3D scene with joints which are represented as blue circles. Figure 13b shows the 3D scene with joints after motion using the partition and non-partition optimization model. As mentioned in above, in these figures, arrows indicate the transformation motion, red circles represent joints after solving with non-partitioned model using COBYLA solver while green circles represent the joints after solving with partitioned model using COBYLA solver. In Fig. 13c the differences using Canny edge detection algorithm were marked with red. In conclusion, our optimization model achieves the desired motion with minimal errors. In conclusion, our optimization model achieves the desired motion with minimal errors in both case studies.

## Conclusion

In this work, we introduced a partition-based solution to our original optimization model for developed GAML. Our approach is based on creating smaller sub-problems of original model with achieving acceptable error rates and practical computation times for increasing number of the constraints. Our goal is to achieve near real time rates to overcome exponential increase in the computation time of our non-linear model. We used COBYLA as non-linear optimization solver. In our approach, we partitioned the problem joints network and introduced new virtual nodes and additional constraints to retain the original problem. Partitioning of the problem was carried out with detecting communities with CNM, k-means and DBSCAN algorithms. Execution of partitioned problems, instead of solving original and large problem, significantly reduces non-linear execution time (up to 2689.7 speed-up for constraint set of 5000) and decreased the error. For the constraint set of 5000 the variation in error was between 3.57 and 2.2%. Lowest error calculated for the constraint set of 5000 was 2.2% error using CNM, while k-means-5 had the highest error percentage at 3.57%. The error percentage for rest of the community detection/clustering algorithms are calculated as DBSCAN-10 - 2.4%, DBSCAN-5 - 2.34% and k-means - 3.2%. We used our approach for modeling virtual 3D arthroscopic rotator cuff and airway anatomy. Our findings showed that errors in our scenes were minimal where we used an edge detection algorithm to visualize them. In the future, we plan to experiment our approach using different joint hierarchy graphs and community detection techniques with higher constraint sets for further validation.

## Abbreviations

(*p*_*i*_ − *p*_*j*_)_*axis*_: Directional vectors in the x, y or z axis’ to accommodate any lock along the axis of rotation
*a*_*ij*_: Angle between joints *J*_*i*_ *and J*_*j*_
*k*_*l*_: Stiffness ratio of joint l
*∆d*_*max*_: Is the maximum displacement allowed between the joint couple *p*_*i*_ and *p*_*j*_
BARON: Branch – And- Reduce Optimization Navigator
*CNM*: Clauset Newman Moore
COBYLA: Constrained Optimization by Linear Approximation
CPOPT: A Partition-and-resolve procedure for solving P_t_
*DBSCAN*: Density-based spatial clustering of applications with noise
*Dist*_*ij*_: The original distance between joint *J*_*i*_ *and J*_*j*_
GAML: Generative Anatomy Modeling Language
GRG: Generalized Reduced Gradient
*J*_*i*_: *Joint i*
LIBSVM: Library for Support Vector Machines
M: 3D mesh
MacMINLP: collection of mixed integer non-linear programs
MINLP: Mixed Integer Non-linear Programming
MINPTS: The amount of joints required for a dense area in DBSCAN
*N*: *Number of joints*
*p*_*i*_: a 3D position of a joint *J*_*i*_
*p*_*io*_: The initial point for joint *p*_*i*_
POM-GAML: Partition-based Optimization Model for Generative Anatomy Modeling Language
*P*_*t*_: Partition t
QP: Quadratic Programming
SMO: Sequential Minimal Optimization
SQP: Sequential Quadratic Programming
SVM: Support Vector Machine
v: Virtual joint
ε: Radius used in DBSCAN
*θ*_*ij*_: Maximum angle that joint *p*_*i*_ is allowed to pivot about joint *p*_*j*_*.*
*r*: Radius of the cone
